# Unveiling the Prevalence and Factors of Workplace Bullying in Primary Healthcare Settings: A Cross-Sectional Study in Jeddah City, Saudi Arabia

**DOI:** 10.7759/cureus.41382

**Published:** 2023-07-05

**Authors:** Suhail M Abdulkarim, Abeer A Subke

**Affiliations:** 1 Preventive Medicine Postgraduate Program, Ministry of Health, Jeddah, SAU

**Keywords:** factors, prevalence, doctors, nurses, healthcare workers, bullying in healthcare, workplace bullying

## Abstract

Introduction

Bullying in workplaces can lead to serious and deleterious effects on both the health and well-being of individuals. In a healthcare environment, bullying can lead to life-threatening adverse outcomes for patients and healthcare workers. The aim of this study was to evaluate the prevalence and factors of bullying among primary healthcare workers in Jeddah, Saudi Arabia.

Methods

This cross-sectional study targeted physicians and nurses in Jeddah healthcare centers and used a Negative Acts Questionnaire-Revised (NAQ-R) to evaluate participants’ exposure to bullying. The chi-square test was used to examine the relationship between the outcome and other variables.

Results

The majority of participants (59.8%) had more than 10 years of experience and were nurses (56.6%). The majority of participants (69.4%) scored below 33 on the NAQ-R scale, while 19.9% scored between 33 and 45, and 10.7% scored over 45. Most perpetrators were references/patients (22.4%), supervisors (19.2%), department managers, or general managers (19.2%). Of all participants, 28.8% had experienced workplace bullying (WPB), and 31.7% witnessed it over the past five years. Being subjected to WPB (*P *< 0.001), being bullied by a manager (*P *< 0.001), and experiencing and witnessing WPB over the past five years (*P *< 0.001) correlated with higher NAQ-R scores. Years of experience were significantly associated with NAQ-R scores (*P* = 0.016).

Conclusions

This study indicates bullying among a third of healthcare workers, mainly perpetrated by patients and managers. Years of experience and manager offenses, experiencing and witnessing WPB were associated with higher bullying rates. Therefore, there is an urgent need for antibullying policies, awareness campaigns, education programs, effective communication, conflict resolution, leadership training, and transparent culture to address this problem.

## Introduction

Bullying is considered a public health problem that spreads globally in different workplaces and can lead to serious and deleterious effects on individuals' health and well-being [1]. Bullying can disrupt the social interaction between people in the same workplace, which causes unhealthy and seemingly unproductive work results and environment [2]. Most studies that were conducted in the last 30 years define bullying as a group of situations where an individual, over some time and repeatedly, is exposed to abuse, social exclusion, harassment, or offenses, which put the person in an asymmetrical position where they are unable to defend themselves from the different negative behaviors [3]. Bullying usually involves acts or verbal comments that could psychologically or *mentally* hurt or isolate a person in the workplace [1,4]. Bullying typically also entails a pattern of actions or repeated instances meant to terrorize, offend, denigrate, or humiliate a specific person or group of people in aggression to assert power [4]. Since there is no clear definition of workplace bullying (WPB), it may also be defined as repeated acts and practices directed at one or more workers unwelcome by the victim, done intentionally or unintentionally, but clearly cause humiliation, offense, and distress, and may interfere with job performance and/or cause unpleasant working environments [1-7]. There is no doubt that bullying can be overt and scary, leading to hostile working environments and physiological and psychological consequences for workers. These unhealthy workplaces result in high costs due to high leave and turnover rates and lower productivity, satisfaction, and morale [4-6]. In the healthcare workplace, bullying can lead to medical errors, negligence, and increased mortality and morbidity [5]. Deficiencies in leadership behavior and work design, low moral standards, and the socially exposed position of the victim has been identified as the main factors of bullying in the workplace [7]. Hierarchy is one of the characteristics of the hospital environment, making it vulnerable to bullying [8,9]. Bullying was reported in a medical center in Iran by 44.4% of the staff [10]. Similarly, a study conducted in surgical specialties in Saudi Arabia showed that 44.3% of consultants were considered bullies against residents and interns [6]. This study also found a significant association between bullying and age, specialty, and position. Alswaid reported that bullying was prevalent against new nurses and other staff and was influenced by the hierarchy (strict chain of command and control) in five hospitals in Riyadh, having an impact on their performances and patients [11]. There are still limited studies conducted in Saudi Arabia exploring WPB in healthcare facilities. Thus, this study determined the prevalence of WPB and the contributing factors among primary healthcare workers.

## Materials and methods

Study design

We conducted a cross-sectional study in Jeddah, Saudi Arabia, from April 1 to May 21, 2023. We included primary healthcare workers (physicians and nurses) in Jeddah healthcare centers with at least six months of working experience to ensure the participants interacted sufficiently with superiors, coworkers, and patients and fulfilled the criteria of at least six months to consider WPB. Those with experience of less than six months, those who were on leave at the time of the study, pharmacists, and allied health personnel were excluded.

Sample size

The sample size of 336 was calculated using Raosoft software (Raosoft Inc., Seattle, WA, USA), taking into account various factors such as a margin of error of 5%, a confidence level of 95%, a total population of 1,500 healthcare workers in Jeddah [12], and an estimated response level of 50%.

Sampling technique

A two-stage cluster sampling technique was used. Jeddah was divided into North and South regions. The healthcare centers were randomly selected from both regions, and participants were also randomly selected from each center.

Data collection tool and procedure

An anonymously self-administered online questionnaire with 30 questions was used for data collection (Appendix). It was composed of three sections: Section 1 had study information and consent; Section 2 collected sociodemographic characteristic data: sex, age, nationality, marital status, education, years of experience, and occupation; and Section 3 used a Negative Acts Questionnaire-Revised (NAQ-R) consisting of 22 questions that reflected the frequency of exposure to negative actions with three subscales, work-related, person-related, and physically intimidating. Responses are based on a five-point scale ranging from never, now and then, monthly, weekly, and daily. Following that, a self-labeling with definition approach is utilized to address numerous questions about the experience of bullying, such as the frequency, the duration of the experience, and who the primary perpetrators are [13,14]. NAQ-R was translated and adapted in Arabic with Cronbach's alpha coefficients ranging from 0.63 to 0.90 [14]. The overall NAQ-R scale ranged from 22 to 110, and respondents' scores were divided into three groups based on their exposure to WPB, using two cutoff points. Those with a score below 33 were classified as never bullied, with a score between 33 and 45 were classified as occasionally bullied, and those with a score above 45 were classified as severely bullied. Similar classifications were used and detailed in previous studies [13,14].

The investigators arranged with each healthcare center's administrator to provide a list of all workers and their mobile phone numbers. Then the questionnaire was distributed via text message as a link together with consent forms.

Statistical analysis

We performed descriptive and analytic statistics using Statistical Package for the Social Sciences SPSS, Version 21 (SPSS, Chicago, IL, USA). We performed descriptive statistics and presented data as mean and standard deviation (SD) or frequency (percentage) for the qualitative variables. The chi-square test was implemented to test the association between the outcome and other variables. The results were evaluated against a confidence interval of 95% and a *P*-value < 0.05 for statistical significance.

Ethical approval

This study was approved by the Research Ethics Committee of the Ministry of Health, Jeddah (Ref. No. A01602) on March 16, 2023.

## Results

This study received 281 responses (83.6%) out of 336 questionnaires sent. Table 1 shows the sociodemographic characteristics of participants. The majority of the participants (67.6%) were in the age group of 30 to 40 years, followed by those aged 41 to 50 years (23.1%). Almost three-quarters were female (74%), and the rest were males. The majority of participants were Saudi nationals (99.6%) and married (80.4%). The majority of the participants (41.6%) held diplomas, followed by bachelor's degrees (37%). The majority of the participants (59.8%) reported having more than 10 years of experience. Most participants (56.6%) were nurses, followed by doctors (34.5%).

**Table 1:** Sociodemographic characteristics.

Characteristics	n	%
Age (years)		
<30	7	2.5
30-40	190	67.6
41-50	65	23.1
51-60	19	6.8
Gender		
Male	73	26
Female	208	74
Social status		
Single	35	12.5
Married	226	80.4
Divorced/Widow	20	7.1%
Nationality		
Saudi	280	99.6
Non-Saudi	1	0.4
Academic qualifications		
Diploma	117	41.6
Bachelor	104	37
Master	17	6
Ph.D./Fellowship	43	15.3
Years of service		
<2	3	1.1
2-10	110	39.1
>10	168	59.8
Job		
Doctor	97	34.5
Dentist	25	8.9
Nurse	159	56.6

On the NAQ-R scale, the majority of participants (69.4%) were never bullied (NAQ-R score < 33), 19.9% were occasionally bullied (NAQ-R score = 33-45), and 10.7% were severally bullied (NAQ-R score > 45) (Table 2).

**Table 2:** Participants' responses on the NAQ-R scale. NAQ-R, Negative Acts Questionnaire-Revised

Classification of NAQ-R score	n	%
Never bullied	195	69.4
Occasionally bullied	56	19.9
Severally bullied	30	10.7
Total	281	100

Table 3 details the personal experience of WPB and witnessing bullying over five years. The majority of respondents (70.1%) reported not being subjected to WPB, 16.7% reported experiencing bullying in rare cases, and 11% reported experiencing it from time to time. Of those who reported experiencing WPB, most (12.1%) indicated that the bullying started between the last six months and 12 months. Most reported being bullied by one to three men (22.4%) and one to three women (19.6%). Almost a quarter (22.4%) reported being bullied by patients, and almost a fifth reported being bullied by supervisors, department managers, or general managers (19.2%). Additionally, smaller proportions reported being bullied by colleagues (8.5%) and employees (3.2%). Over the past six months, most (13.9%) witnessed WPB from time to time, followed by those who witnessed it occasionally (6.8%). However, 28.8% reported having experienced WPB over five years and 31.7% reported witnessing such incidents.

**Table 3:** Participants’ experience regarding workplace bullying.

Exposure to workplace bullying	n	%
Are you subjected to workplace bullying?		
No	197	70.1
Yes, in rare cases	47	16.7
Yes, from time to time	31	11
Yes, several times a week	4	1.4
Yes, almost daily	2	0.7
When did the bullying start?		
During the past six months	18	6.4
Between the last six months and 12 months	34	12.1
Between one and two years	12	4.3
More than two years ago	20	7.1
How many men have bullied you?		
0	10	3.6
1-3	63	22.4
4-6	5	1.8
>7	6	2.1
How many women have bullied you?		
0	21	7.5
1-3	55	19.6
4-6	8	2.8
Have the supervisor or departmental manager or general manager bullied you?		
No	29	10.3
Yes	54	19.2
Have a colleague or colleagues bullied you?		
No	59	21
Yes	24	8.5
Have employees or employees bullied you?		
No	74	26.3
Yes	9	3.2
Have patients bullied you?		
No	20	7.1
Yes	63	22.4
How many people have been bullied?		
Just you	25	8.9
You and several other coworkers	56	19.9
Everyone in the working group	3	1.1
Have you witnessed bullying in your workplace over the past six months?		
No, never	15	5.3
Yes, but rarely	19	6.8
Yes, but time to time	39	13.9
Yes, often	11	3.9
Have you ever experienced workplace bullying over the past five years?		
No	200	71.2
Yes	81	28.8
Have you witnessed workplace bullying over the past five years?		
No	192	68.3
Yes	89	31.7

Table 4 presents the association between the NAQ-R scores of participants' outcomes and various sociodemographics. Only years of experience demonstrated a significant positive association with NAQ-R scores (*P* = 0.016). Participants with two to 10 years and more than 10 years of experience reported significantly more bullying than those with less than two years of experience. There were no significant associations with the remaining sociodemographics (*P *> 0.05).

**Table 4:** Association between NAQ-R scores and sociodemographics. *P *< 0.05: statistically significant. NAQ-R, Negative Acts Questionnaire-Revised

Demographics	Never bullied		Occasionally bullied		Severally bullied		*P*-value
	n	%	n	%	n	%	
Age (years)							
<30	6	3.1	0	0.0	1	3.3	0.872
30-40	131	67.2	38	67.9	21	70.0	
41-50	44	22.6	15	26.8	6	20.0	
51-60	14	7.2	3	5.4	2	6.7	
Gender							
Male	49	25.1	15	26.8	9	30.0	0.842
Female	146	74.9	41	73.2	21	70.0	
Social status							
Single	22	11.3	5	8.9	8	26.7	0.043
Married	162	83.1	46	82.1	18	60.0	
Divorced/Widow	11	5.6	5	8.9	4	13.3	
Nationality							
Saudi	195	100.0	55	98.2	30	100.0	0.133
Non-Saudi	0	0.0	1	1.8	0	0.0	
Qualifications							
Diploma	90	46.2	19	33.9	8	26.7	0.127
Bachelors	72	36.9	21	37.5	11	36.7	
Master	9	4.6	5	8.9	3	10.0	
Ph.D./Fellowship	24	12.3	11	19.6	8	26.7	
Years of experience							
<2	3	1.5	0	0.0	0	0.0	0.016
2-10	65	33.3	26	46.4	19	63.3	
>10	127	65.1	30	53.6	11	36.7	
Job							
Doctor	65	33.3	20	35.7	12	40.0	0.209
Dentist	13	6.7	7	12.5	5	16.7	
Nurse	117	60.0	29	51.8	13	43.3	

As indicated in Table 5, the more subjected to WPB, the more participants experienced bullying (the higher NAQ-R scores) (*P *< 0.001). Being bullied by a manager significantly correlated with a high frequency of experiencing bullying (high NAQ-R scores) (*P *< 0.001). Experiencing and witnessing WPB over the past five years was also significantly associated with more incidences of being bullied (high NAQ-R scores) (both *P *< 0.001).

**Table 5:** Association between NAQ-R scores and participants’ experience regarding workplace bullying. *P *< 0.05: Statistically significant. NAQ-R, Negative Acts Questionnaire-Revised

Classification of NAQ-R scores	Never bullied		Occasionally bullied		Severally bullied		*P*-value
	n	%	n	%	n	%	
Are you subjected to workplace bullying?							
No	169	86.7	23	41.1	5	16.7	<0.001
Yes, in rare cases	21	10.8	18	32.1	8	26.7	
Yes, from time to time	5	2.6	14	25	12	40	
Yes, several times a week	0	0.0	1	1.8	3	10	
Yes, almost daily	0	0.0	0	0.0	2	6.7	
When did the bullying start?							
During the past six months	8	30.8	4	12.1	6	24	0.617
Between the last six months and 12 months	11	42.3	13	39.4	10	40	
Between one and two years	3	11.5	6	18.2	3	12	
More than two years ago	4	15.4	10	30.3	6	24	
How many men have bullied you?							
0	5	19.2	2	6.1	3	12	0.783
1-3	18	69.2	26	78.8	19	76	
4-6	1	3.8	2	6.1	2	8	
>7	2	7.7	3	9.1	1	4	
How many women have bullied you?							
0	10	38.5	6	18.2	5	20	0.246
1-3	14	53.8	25	75.8	16	64	
4-6	2	7.7	2	6.1	4	16	
Have the supervisor or departmental manager or general manager bullied you?							
No	16	61.5	10	31.3	3	12	<0.001
Yes	10	38.5	22	68.8	22	88	
Have a colleague or colleagues bullied you?							
No	23	88.5	24	75	12	48	0.005
Yes	3	11.5	8	25	13	52	
Have an employee or employees bullied you?							
No	26	100	29	90.6	19	76	0.021
Yes	0	0.0	3	9.4	6	24	
Have references or patients bullied you?							
No	7	26.9	8	25.0	5	20	0.836
Yes	19	73.1	24	75.0	20	80	
How many people have been bullied?							
	9	34.6	10	30.3	6	24	0.946
You and several other coworkers	16	61.5	22	66.7	18	72	
Everyone in the working group	1	3.8	1	3.0	1	4	
Have you witnessed or been a witness to bullying occurring in your workplace over the past six months?							
No, never	8	30.8	5	15.2	2	8	0.232
Yes, but rarely	6	23.1	7	21.2	6	24	
Yes, but from time to time	10	38.5	18	54.5	11	44	
Yes, often	2	7.7	3	9.1	6	24	
Have you ever experienced workplace bullying over the past five years?							
No	167	85.6	27	48.2	6	20	<0.001
Yes	28	14.4	29	51.8	24	80	
Have you witnessed workplace bullying over the past five years?							
No	163	83.6	22	39.3	7	23.3	<0.001
Yes	32	16.4	34	60.7	23	76.7	

## Discussion

WPB is a widespread problem affecting employees in various industries, including healthcare [15]. Bullying undermines the welfare of healthcare professionals and jeopardizes patients' safety in the healthcare context, where personnel are entrusted with the care and well-being of others. This study aimed to evaluate the prevalence of healthcare WPB and identify associated factors. The findings could help raise awareness and develop strategies and interventions to effectively address and prevent WPB in healthcare environments.

Our study showed that 30% of participants were bullied, aligning with previous studies reporting a WPB prevalence of 20% to 30% among healthcare workers [2,15,16]. Among nurses, a previous study in Saudi hospitals showed that WPB was 30% more prevalent among younger nurses and less prevalent among higher-educated nurses [17]. Among physicians, it was 33% more prevalent among females, and among those, it was more prevalent among higher-educated physicians (47%) [17]. Research indicated that healthcare workers, doctors, nurses, and support personnel face higher rates of WPB than workers in other industries [15,16]. Regarding sex, a survey in the United States indicated that two-thirds of men are the bully [18], which is consistent with what our study showed. In academic medicine, it was also found that men are the most common bullies [19], which is similar to the findings among National Health Service (NHS) staff in the United Kingdom [20]. Patients were the most common perpetrators, followed by supervisors and managers. Another previous study conducted among surgical teams in Saudi Arabia showed consultants to be the most offenders [6]. Another study conducted in multiregional Saudi hospitals showed that perpetrators were mainly patients (36.1%), followed by their families/relatives (29.5%) and hospital staff (27.2%) [17]. Similarly, previous studies showed that patients and employees in leadership positions are the most perpetrators [18]. The hierarchical nature of healthcare institutions has been found to contribute to bullying, with those in high positions being the most bullying against other employees, such as nurses and students [8]. This might be attributed to high-stress environments and long hospital working hours. High workload and pressure also contribute to stress and tensions within the workplace, increasing the likelihood of bullying.

Our study showed that being bullied by a manager was associated with high NAQ-R scores (*P *< 0.001), showing the influence of hierarchy. This may be attributed to the power imbalances stemming from hierarchical structures, and a culture of deference to authority can enable bullying behaviors [21]. It was found that bullying victims were mostly those who work on a shift schedule, rotating performers, work stress sufferers, and those dissatisfied with their working conditions [15]. A previous study in tertiary care hospital in Riyadh showed that the prevalence of bullying was positively correlated with lower job satisfaction and mental health levels [22]. In Norway, bullying against nurses by patients and their next of kin was found to be more common than WPB [23], aligning with our study findings. Nurses were reported vulnerable to bullying, which might be due to being in front of patient care and their frequent encounters with coworkers and superiors [23,24]. Moreover, most nurses in Saudi Arabia are women, who are more vulnerable to bullying [18,25]. Though our study found no significant association between NAQ‑R scores and age, specialty, and position, as reported among surgical staff in Saudi Arabia by Albuainain et al. [6], it showed a significant association with years of experience (*P *= 0.016). This is probably from the fact the more years spent in a workplace, the more likely they are to experience bullying, and our study involved nurses and doctors in different specialties, while the study by Albuainain et al. included surgical staff only. This is also supported by our findings that experiencing or witnessing bullying over the last five years was associated with NAQ-R scores (*P *< 0.001). Bullying can manifest in various forms, such as verbal abuse, humiliation, intimidation, ostracism, and sabotage. In Saudi hospitals, research showed that verbal abuse was the most common type of WPB incident reported, followed by physical harassment and sexual connotations [17]. Studies indicated that organizational problems such as a lack of workplace antibullying policies, insufficient training on conflict resolution and communication skills, and poor leadership support could worsen the problem [26].

There are some limitations for consideration. This study was cross-sectional, prone to selection bias, and limited in making causal inferences. This study excluded other professionals working in healthcare facilities and students whose services also affect patients and could be jeopardized by bullying. It also excluded participants with less than six months of experience, missing their reports about bullying they might have experienced. The online design is also prone to underreporting or overreporting by participants. Self-administered questionnaires rely on a person's interpretation of the term "bullying", which may lead to some under-reporting and over-reporting. We recommend prospective studies including a large sample of different professionals to show a wide picture of bullying and associated factors in Saudi Arabia.

## Conclusions

This study showed bullying in 30% of studied healthcare workers, perpetrated mainly by patients and managers of the hospitals. Years of experience and offense by managers were positively associated with the frequency of being bullied. Hospitals in Jeddah should tackle this problem by implementing targeted measures to re-inform policies designed to create a friendly working environment, respect, and professionalism. Further studies are also recommended to explore bullying from patients to inform preventive measures.

## Appendices

Nَّegative Acts Questionnaire-Revised: Arabic version

إن السلوكيات أدناه غالباً ما تعتبر مثاال العمال سلبية في مكان العمل.

خالل األشهر الستة الماضية، ما مدى تكرار تعرضك لهذه االعمال السلبية ؟

رجاءً ضع دائرة حول الرقم الذي يتطابق اكثرما يمكن مع تجربتك في مكان عملك خالل األشهر الستة الماضية.

1

2

3

4

5

6

7

8

9

1

2

3

4

ً

من حين الى أخر

ً

ً

أبدا

شهريا

أسبوعيا

هناك من يخفي معلومات تؤثّر على أدائك

تعرّضت لإلهانة اوالسخرية بما يتعلق في عملك

ُأمرت بالقيام بعمل دون مستوى كفاءتك

تمّت ازالة أو استبدال نقاط رئيسيّة من مسؤولياتك بمهام أخرى تافهة أو مزعجة

هناك ثرثرة أو نشر اشاعات عنك

تمّ تجاهلك او استبعادك، او مقاطعتك

ِوجّهت لك مالحظات مهينة أو مسيئة عن شخصيتك )مثالً عاداتك و خلفيتك( أو مواقفك أو حياتك الخاصة

تمّ الصراخ عليك أو كنت هدفا لفورات غضب تلقائية

تعرّضت لسلوكيات تهويل مثل هزّ االصبع، اقتحام مساحتك الشخصية، الدفع، أوصدّ الطريق

5

يوميا

1

2

3

4

5

1

2

3

4

5

1

2

3

4

5

1

2

3

4

5

1

2

3

4

5

1

2

3

4

5

1

2

3

4

5

1

2

3

4

5

1

2

3

4

5

10

وجهت لك تلميحات أو اشارات من الغير انه عليك االستقالة من وظيفتك

1

2

3

4

5

11

تعرّ

ضت لتذكيرات متكررة بأخطائك أو أغالطك

1

2

3

4

5

21                   تمّ تجاهلك أو واجهت ردة فعل عدائيّة لدى اقترابك من اخرين

31                تعرّضت النتقادات مستمرة حول عملك ومجهودك

41                   تمّ تجاهل أرائك وأفكارك

51       تعرّضت لمقالب من قبل أشخاص ال تتفق معهم

61        تمّ إعطاُؤك مهمّات النهائها بمهلة زمنية غير معقولة أو مستحيلة

71       تمّ توجيه االدعائات ضدك

81       تعرّضت لمراقبة مفرطة لعملك

91       تمّ الضغط عليك كي ال تطلب شيئاً لك الحق فيه )مثل اجازة مرضية، استحقاق عطلة، تكاليف سفر(

2         تعرّضت إلغاظة أو لسخرية مفرطة

12                تعرّضت لعبئ عمل ال يمكن إدارته

22                تعرَّضت لتهديدات بالعنف أو االعتداء الجسدي أو الساءة فعلية

1    5    4     3     2

1    5    4     3     2

1    5    4     3     2

1    5    4     3     2

1    5    4     3     2

1    5    4     3     2

1    5    4     3     2

1    5    4     3     2

1    5    4     3     2

1    5    4     3     2

1    5    4     3     2

.23 هل تتعرَّض للتنمُّر في العمل ؟

يعرِّف الُّتنمر (Bullying)على أنه وضع يتصوّر فيه فرد أو عدة افراد أنهم موضع تلّّقي اعمال سلبية من قبل واحد أو عدة أشخاص و ذلك باستمرار وعلى مدى فترة من الزمن، و يكون فيها المستهدفون من التنمُّر في حالة صعوبة في الدفاع عن نفسهم ضد هذه األفعال.

و ال ُيَوصّف الحادث االستثنائي الذي يقع لمرة واحدة فقط كتنمُّر.

مستخدماً هذا التعريف، يرجى االشارة ما إذا كنت تعرضت للتنمُّر في مكان عملك خالل األشهر الستة الماضية؟

كال، )اذهب الى السؤال ) 29 نعم، في حاالت نادرة نعم، بين الحين واآلخر

نعم، عدة مرات في األسبوع نعم، بشكل شبه يومي

42.   متى بدأ التنمُّر؟

خالل األشهر الستة الماضية

بين 6 اشهر و 12 شهر الماضيين بين السنة و السنتين الماضيتين منذ أكثر من سنتين

52.            كم عدد األشخاص الذين َّتنمرواعليك؟ عدد الرجال: ___________

عدد النساء: ___________

62.              من تنمّر عليك؟ )يمكنك وضع عالمة على أكثر من احتمال واحد( مشرف، مدير قسم ، مدير عام زميل \ زمالء موظف \ موظفين تابعين لك

زبون \ زبائن ، تلميذ \ تالميذ

72.              كم شخص تعرّض للُّتنمر ؟ فقط أنت

انت وعدة زمالء أخرين في العمل الجميع في مجموعة العمل

82.              هل رأيت أو كنت شاهداً على التنمّر يحصل في مكان عملك على مدى الستة أشهر الماضية؟ كال، أبداً نعم، لكن

نعم، من الحين الى اآلخر نعم، غالباً

92.              هل تعرّضت يوماً للتنمّر في العمل على مدى السنوات الخمس الماضية؟ كال نعم

30 هل شهدت على التنمّر في مكان عملك على مدى السنوات الخمس الماضية؟

لاك

معن
